# Application of a triblock copolymer additive modified polyvinylidene fluoride membrane for effective oil/water separation

**DOI:** 10.1098/rsos.171979

**Published:** 2018-05-09

**Authors:** S. S. Shen, K. P. Liu, J. J. Yang, Y. Li, R. B. Bai, X. J. Zhou

**Affiliations:** 1 Center for Separation and Purification Materials and Technologies, Suzhou University of Science and Technology, 1 Kerui Road, Suzhou 215009, People's Republic of China; 2 Suzhou Key Laboratory of Separation and Purification Materials and Technologies, Suzhou University of Science and Technology, 1 Kerui Road, Suzhou 215009, People's Republic of China; 3 Jiangsu Collaborative Innovation Center for Technology and Material of Water Treatment, Suzhou University of Science and Technology, 1 Kerui Road, Suzhou 215009, People's Republic of China

**Keywords:** modified PVDF hollow fibre membrane, triblock copolymer additive, hydrophilic and oleophobic properties, oil/water separation

## Abstract

A hollow fibre membrane was fabricated by blending polyvinylidene fluoride (PVDF) with a triblock copolymer additive polymer that has both hydrophilic and oleophobic surface properties. The novel membrane was characterized and examined for oil/water separation under various system conditions, including different cross-flow rate, feed temperature, trans-membrane pressure, and its rejection and cleaning efficiency, etc. By applying the membrane into the filtration of synthesized oil/water emulsion, the membrane constantly achieved an oil rejection rate of above 99%, with a relatively constant permeate flux varied in the range of 68.9–59.0 l m^−2^ h^−1^. More importantly, the fouling of the used membrane can be easily removed by simple water flushing. The membrane also demonstrated a wide adaptability for different types of real oily wastewater, even at very high feed oil concentration (approx. 115 000 mg l^−1^ in terms of chemical oxygen demand (COM)). Hence, the novel triblock copolymer additive-modified PVDF membrane can have a great prospect in the continuing effort to expand the engineering application of polymeric membranes for oily wastewater treatment.

## 1. Introduction

Oily wastewater produced from many industries, such as oil and gas, petrochemical, metallurgical, food and plant oil industries, is a significant source of water pollution. A large amount of harmful oily wastewater is discharged every year [1]. Traditional treatment methods for oily wastewater include gravity settling, dissolved air flotation, chemical coagulation and flocculation, etc. [2]. These methods are usually limited to the removal of free and dispersed oil that has larger oil droplet sizes (greater than 20 µm), but they are often hard or costly to use for removal of emulsified oil with much smaller droplet sizes (much less than 20 µm) [3].

Membrane separation technology has a good perspective for the purification of water/oil emulsions due to its many advantages, such as simple system, short process time, high separation efficiency and no chemical consumption necessary [4–6]. However, most membranes in the market are polymeric and they are unable to be effectively used for oil/water separation, because of the severe membrane fouling caused by rapid adsorption or adhesion of oil droplets on the surfaces or internal pores of the membranes, which may diminish membrane filtration performance and its productivity in a very short period of time or even within seconds (if oil concentration is high). Furthermore, oily foulants often cause irreversible fouling to these membranes, leading to high operational cost and complex system operation strategies [7]. Developing highly effective anti-oil-fouling membranes has, therefore, long been desired. A large number of investigations have found that increasing membrane's surface hydrophilicity can reduce membrane fouling [8–17]. However, those hydrophilic membrane surfaces usually have a relatively high surface free energy (much higher than the surface tension of oils but often lower than that of water). Thus, oils not only have a strong tendency to attach to the membrane surfaces, but, once attached, also become more difficult to remove by normal membrane cleaning [18]. Therefore, it has been envisioned that the hydrophilic membrane modification approach cannot completely avoid membrane fouling, especially the one caused by oils [19].

A suitable membrane surface for oil/water separation should ideally bear not only hydrophilic but also oleophobic properties [20–23]. The hydrophilicity helps the membrane to achieve higher water permeability or water flux with lower resistance, while the oleophobicity can offer the membrane a very low apparent surface free energy (lower than that of oils), thus realizing low or no adhesion of oils, due to the weak interaction strength between the membrane surface and the foulants, such as oils [24]. In this line, some studies prepared amphiphilic polymers with hydrophilic and oleophobic groups for surface modifications. Howarter & Youngblood reported the preparation of a surfactant containing perfluorinated end (oleophobic) and polyethylene glycol chain (hydrophilic) that was covalently grafted onto glass membranes for oil/water separation performance [25–27]. A nano-composite PDDA-PFO prepared from poly(diallyldimethylammonium chloride) (PDDA) and sodium perfluorooctanoate (PFO) was coated onto stainless steel mesh and it was reported to show simultaneous superhydrophilic and superoleophobic properties [28]. Yang *et al.* reported a membrane fabricated by grafting hyperbranched poly(ethylene imine) onto polyvinylidene fluoride (PVDF) and polyacrylic acid-grafted PVDF blend membrane and showed the modified membrane surface exhibiting superhydrophilicity, and, in acidic conditions, superoleophobicity for oily wastewater treatment [29]. However, all these studies were limited on small flat membrane films for laboratory tests, which may face the challenge of scale-up needed for practical engineering applications. A grafted triblock copolymer with superhydrophilicity and superoleophobicity was synthesized by Bai and co-workers [20,30,31]. The synthesized polymer was used as an additive polymer (AP) and blended with the base membrane material PVDF. Both flat-sheet membrane and hollow fibre membrane samples were prepared and tested in the laboratory and the results indicated very good performance in the treatment of oily wastewater.

In this study, similar method as reported by Bai and co-workers was used to prepare hollow fibre membranes with both hydrophilic and oleophobic surface properties through blending the triblock copolymer additive with PVDF in certain specific ratios. The hollow fibre membranes were further packed into membrane modules and were operated under the cross-flow filtration mode for oil-in-water emulsion separations. The performance of the hollow fibre membrane was examined under various operational conditions and for different types of synthetic as well as real oily wastewater samples.

## 2. Material and methods

### 2.1. Materials

The triblock copolymer additive, P(VDF-co-CTFE)-g-PMAA-g-fPEG (denoted as AP), was synthesized via the multi-step reactions [20]. Polyvinylidene fluoride (PVDF, MW approx. 322 000) was dried at 100°C for 24 h before use. 1-Hexadecane, *N*-methyl-2-pyrrolidine (NMP) and other chemicals used in this study were all purchased with analytical quality and used as received. Deionized (DI) water (18.2 MΩ) purified with a Milli-Q system from Millipore was used to prepare all solutions as needed in the work.

### 2.2. Preparation of hollow fibre membranes

PVDF and AP were blended in a certain weight ratio and dissolved in NMP to prepare the dope solutions for spinning the hollow fibre membranes. The total polymer concentration in the dope was at 16 wt%. The fabrication of the hollow fibre membrane was carried out by the common dry–wet spin phase inversion method [32]. In brief, the blended mixture of PVDF and AP in NMP was heated and stirred at 80°C to give a clear homogeneous solution. The solution was degassed under a slight vacuum at 80°C for 24 h. The dope solution was then extruded through a spinneret (with outer and inner diameters at 1.5 and 0.9 mm, respectively) into an external coagulation bath. Bore coagulation liquid was also supplied simultaneously on the lumen side of the hollow fibre. Both liquids in the coagulation bath and in the bore were DI water at 45°C. The spun hollow fibre membrane was kept in the coagulation bath for 24 h, and then transferred into another tank with 35 wt% glycerine aqueous solution for at least 12 h. Finally, the membrane was washed with water, dried naturally at ambient temperature and used for characterization analysis or for package into hollow fibre membrane modules for filtration tests.

### 2.3. Characterization of hollow fibre membranes

#### 2.3.1. Surface wetting properties

The surface wetting properties were evaluated by measuring the water and oil surface contact angles on each type of flat-sheet membranes prepared from the same blended polymer mixtures used for the corresponding hollow fibre membranes under the same conditions. A contact angle goniometer (Ramé-Hart 500) was used for the surface contact angle measurements. In brief, a flat-sheet membrane sample was first carefully placed on the horizontal platform of the instrument, with the feed side surface up. A 4 µl droplet of DI water or 1-hexadecane oil was dropped onto the feed side of the membrane surface. The droplet image was captured and analysed by the instrument to obtain the water or oil contact angle value of the tested membrane. Each sample was measured 10 times at different locations on the surface and the average value of these measurements was reported as the representative value in this paper. The corresponding errors (standard deviations) were also calculated and are presented.

#### 2.3.2. Water permeability and the pore size measurement

Water permeability of hollow fibre membranes was tested by using a custom-made filtration system operating in the cross-flow mode. All membrane samples were pressurized with DI water at the average trans-membrane pressure (TMP) of 0.1 MPa for 30 min until membrane performance was basically stable. The permeation flux (*J*) was estimated by the following equation: *J* = *V*/(*A* × Δ*t*), where the parameters *V*, *A* and Δ*t* were defined as the DI water or permeate volume (l), membrane area (m^2^) and permeation time (h), respectively. Each sample was measured three times with the average values as the membrane water flux. The errors (standard deviations) were also calculated.

The maximum pore size of the hollow fibre membranes was measured according to the bubble point method [33] by following the equation: *d* = 4*δ*cos *θ*/Δ*P*, where *δ* is the surface tension of water (mN m^−1^), *θ* is the water contact angle of the membrane (°) and Δ*P* is the bubble point pressure (MPa). Each hollow fibre membrane was tested at least three times, the average data and the errors (standard deviations) were determined and are shown in the paper.

#### 2.3.3. Mechanical properties

The mechanical properties of the prepared hollow fibre membranes were measured with an advanced mechanical testing system, INSTRON 5944. A dry hollow fibre membrane sample was cut into a 15 cm length piece and vertically attached to the two clamps of the machine to give an initial gauge length of 10 cm. The dragging rate of the grip was set at 1 cm min^−1^. The tensile stress and elongation at breakage of the hollow fibre membrane were obtained by the instrument. At least six samples were tested for each type of the hollow fibre membranes, with the average value being reported for it in this paper. The standard deviations were also determined.

#### 2.3.4. Membrane morphology

The surfaces and cross sections of the hollow fibre membrane samples were observed with a scanning electron microscope (SEM, Phenom Pro). The membrane samples were freeze-fractured in liquid nitrogen to produce clean and regular cross sections. The SEM images were then obtained by following the standard operation procedures of the microscope.

### 2.4. Oil/water separation experiments

#### 2.4.1. Preparation of membrane module

The hollow fibre membrane modules were prepared and consisted of a Plexiglas tube (ID = 45 mm) with an effective length of 270 mm. Within the tube, 300 hollow fibres of the same length as the tube were installed to work in parallel. The total membrane filtration area in the membrane module was 0.39 m^2^. The filtration direction was from outside to inside of the hollow fibres in this study. Each membrane module was first immersed in DI water for 24 h and then filtrated with DI water under 1 bar pressure for 2 h before it was used for the oil/water separation tests in the study.

#### 2.4.2. Oil/water emulsion

Synthetic oil/water emulsion samples were first prepared with 1-hexadecane and DI water for the filtration tests. A 400 mg l^−1^ oil concentration in the emulsion was obtained by homogenizing 4 g of 1-hexadecane in 10 l DI water at 10 000 r.p.m. by a homogenizer for 30 min. The oil droplet size distributions in the prepared sample were characterized with a particle counter (Zeta PALS) which can provide the size readings in channels between 0.3 nm and 3.0 µm. As shown in electronic supplementary material, figure S1, the size distribution of the oil droplets for the prepared 400 mg l^−1^ oil/water emulsion was in a very narrow size range, centred at approximately 1.26 µm. The prepared oil/water emulsion samples appeared to be very stable and there was no obvious phase separation observed during the entire filtration experiments.

#### 2.4.3. Filtration separation experiments

All the experiments were conducted in the cross-flow filtration mode. For a specific experimental run, the hollow fibre membrane module was operated under a constant TMP. It was first filtered with DI water for half hour and the stabilized permeate flux was recorded as *J*_0_ (l m^−2^ h^−1^). Then, the feed of the membrane module was switched to the oil/water emulsion sample prepared in another feed tank and the filtration separation test was continued for 2 h. The changes of the permeate flux versus filtration time were calculated on every 5 min interval scale from the recorded permeate weights within the 5 min. The final permeate flux at the end of 2 h filtration was determined and denoted as *J*_p_ (l m^−2^ h^−1^). The relative flux decay (RFD) for each filtration run was hence calculated by RFD = [(*J*_0_ − *J*_P_)/*J*_0_] × 100%.

Membrane cleaning was normally conducted after each filtration run by flushing the membrane surfaces with DI water for 30 min. Sometimes, the cleaning was also followed by flushing the membrane surface with a surfactant solution (sodium dodecyl benzene sulfonate, SDBS) for another 30 min to compare the cleaning efficiency. The permeate flux after each cleaning procedure was measured again with DI water as for *J*_0_ mentioned early and was denoted as *J*_c_. The flux recovery rate (FRR) of the membrane module after cleaning was thus quantified by FRR = (*J*_c_/*J*_0_) × 100%.

The oil concentrations in the feed (*c*_0_) and in the permeate (*c*_p_) of the membrane system were estimated and quantified by total organic carbon (TOC) analysis (TOC-LCPH, SHIMADZU). The oil rejection rate (*R*) of the membrane filtration system was then calculated by *R* = (1 − *c*_p_/*c*_0_) × 100%.

In some later experiments, real oil/water emulsion wastewaters were also collected from related industries and tested by the prepared membrane module. Their oil concentrations were determined by chemical oxygen demand (COD) analyser (DR1010, HACH), each sample was tested at least three times, the average data were reported with less than 5% errors (see details in §3.4).

## 3. Results and discussion

### 3.1. Properties of the prepared hollow fibre membranes

**Figure 1.**
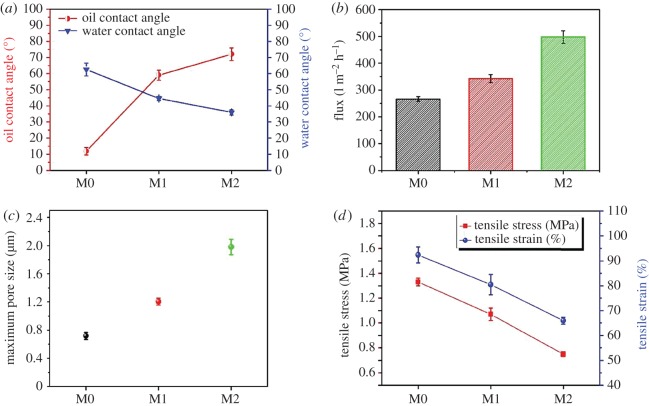
Properties of the blended membranes in the PVDF/AP ratio of 10 : 0 (M0), 9 : 1 (M1) and 8 : 2 (M2).

Blended membranes were fabricated in different ratios of the matrix polymer PVDF to the AP, denoted as M0 (10 : 0, no additive), M1 (9 : 1) and M2 (8 : 2), respectively, in this study. Both hollow fibre membranes and flat-sheet membranes in the same composition were obtained to study their properties. As illustrated in figure 1*a*, by adding the AP into PVDF, the oil contact angle of the membranes was greatly enhanced from 11.9° for M0 to approximately 59.06° for M1 and even 72.06° for M2. By contrast, their water contact angles were significantly reduced from 62.58° for M0 (PVDF) to 44.63° for M1 and 35.94° for M2. The results indicate that the blended membranes became more oleophobic as well as hydrophilic, which is a desirable property for oil/water filtration membrane. The unique property of the obtained novel membranes can be attributed to the blended AP that has a triblock structure containing PVDF + hydrophilic intermediate segment (PEG) + oleophobic terminal segment (perfluoroalkyl groups) within it. It appeared that the hydrophilic and oleophobic segments of the AP may be enriched on the surfaces of the blend membranes, resulting in the enhanced oleophobic as well as hydrophilic surface properties of the prepared membranes.

The improved hydrophilicity of the prepared membranes can be beneficial for water permeation through the membranes. As can be found in figure 1*b*, the DI water flux was indeed increased from 266.6 l m^−2^ h^−1^ for M0 to as high as 497.6 l m^−2^ h^−1^ for M2 (under TMP of 0.1 MPa). This may also be attributed to the changes in the membrane pore sizes. It was observed that with the increase of AP blended with PVDF, the pore sizes of the obtained membranes became larger (figure 1*c*). This was probably due to a decrease in the compatibility of the AP and PVDF polymers. When the membrane mechanical properties were analysed, it was found that both the tensile stress and the tensile strain of the prepared membranes gradually decreased with the increase of the AP content in the membrane (figure 1*d*). The obtained novel hollow fibre membranes seem to be ideal for the application of low pressure operation, such as that in the case of microfiltration or ultrafiltration.

Although the membranes fabricated with a higher ratio of AP showed better surface properties, the mechanical property is also a very important factor to be considered for practical application in water or wastewater treatment, because the TMP acting on the hollow fibre membrane may cause deformations of the membrane structure [34]. In consideration of both, the hollow fibre membrane M1 with the blend ratio of 9 : 1 for PVDF : AP was specifically selected for the further detailed studies in the following sections.

**Figure 2.**
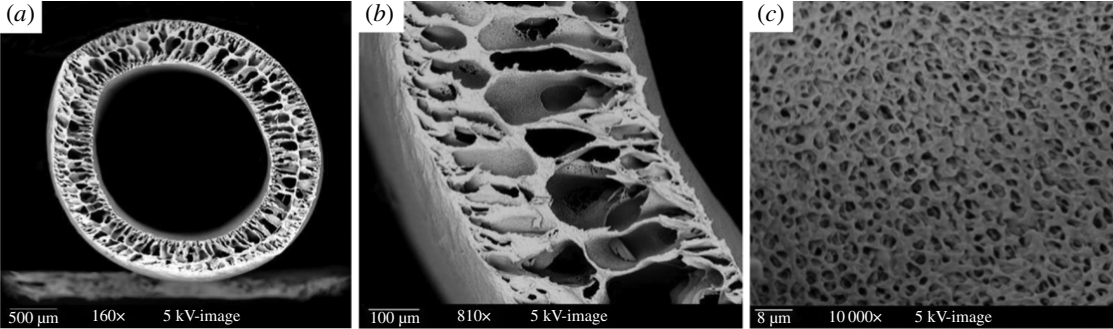
The SEM images of the hollow fibre membrane M1: overview (*a*) and partial view (*b*) of cross-sectional membrane; (*c*) porous microstructure.

The SEM images of the prepared hollow fibre membrane M1 are shown in figure 2. Finger-like macro-voids were observed for the hollow fibre membrane across the cross section, which are typically formed due to delayed liquid–liquid demixing in the coagulation process during the fabrication of the hollow fibre membrane, normally attributed to a lower phase inversion rate of the dope solution [35]. The prepared membrane had a dense outer surface layer supported on the more porous cross-section structure. The highly porous cross-section support is beneficial because it can greatly reduce the fluid resistance of filtration, and easily washed away any contaminants that have passed through the feed surface into the membrane structure. The results in figure 2 also indicate that the novel triblock copolymer additive AP in this study was reasonably compatible with PVDF and hence they formed adequate spinning dope solution and thus uniform microstructure of the hollow fibre membrane. This may be attributed to the PVDF segment at one end of the AP structure, which enhanced the compatibility of AP with the base PVDF membrane material.

### 3.2. Optimization of operation conditions for oil/water emulsion separation

**Figure 3.**
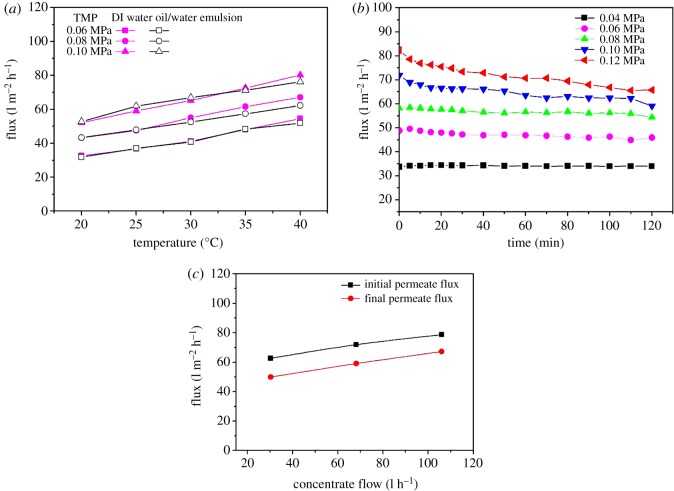
Experimental results of oil/water emulsion filtration, *c*_0_ = 400 mg l^−1^. (*a*) Influence of feed temperature on permeate flux under a TMP of 0.06, 0.08 or 0.10 MPa (cross-flow rate of 68.1 l h^−1^), (*b*) variation of permeate flux versus filtration time under different TMPs (25°C, cross-flow rate of 68.1 l h^−1^) and (*c*) the initial and final permeate fluxes after 2 h filtration under different cross-flow rates (25°C, TMP of 0.10 MPa).

With the prepared hollow fibre membrane M1 module, the operation conditions in oil/water separation performance were studied under a cross-flow filtration mode, including the influences of feed temperature, TMP difference and concentrate flow (or cross-flow) rate. The experimental data are summarized in figure 3.

In oil/water separation, the temperature can often play an important role in the performance. Figure 3*a* shows the results of the influence of feed temperature in the range of 20–40°C under various TMP of 0.06, 0.08 and 0.10 MPa, respectively, with a fixed cross-flow rate of 68.1 l h^−1^ and a feed oil concentration of 400 mg l^−1^. The results of permeate fluxes for both DI water and oil/water emulsion were obtained after a 2 h filtration. It appears that the relationships between the flux and the feed temperature all linearly increased in the examined temperature range, consistent with the results reported in other investigation [36]. This may be mainly due to the viscosity of the feed stream that also almost linearly reduced with the increase of the temperature. There appeared to be only marginal differences in the trend for DI water filtration as compared to that for the oil/water emulsion filtration, the latter of which showed a slightly lower increase. The results suggest that the oil/water emulsion may only have an insignificantly different behaviour to water when the feed temperature changed under the current test conditions.

Generally, increasing the temperature will increase the diffusion coefficients of both the solute and water molecules, and decrease the feed viscosity, therefore leading to the improvement of membrane permeate flux [37,38]. This phenomenon was indeed observed in the study, as shown in figure 3*a*. However, under each TMP, there was no significant difference in the membrane permeate flux for DI water and the oil/water emulsion. It can, therefore, be concluded that the prepared hollow fibre membrane in this study could reduce and even avoid the adsorption of oil onto membrane surface and thus prevent oil fouling effectively.

The permeate flux decay during 2 h oil/water emulsion filtration under different TMPs from 0.04 to 0.12 MPa (at 25°C, cross-flow rate of 68.1 l h^−1^ and feed oil concentration *c*_0_ at 400 mg l^−1^) is shown in figure 3*b*. As can be expected, the permeate flux can be increased by the increase of TMP. Under a higher TMP of 0.12 MPa, for example, the initial flux was more than twice as large as that at the TMP of 0.04 MPa. It is interesting to note that the permeate fluxes at 0.04, 0.06 and 0.08 MPa were almost constant through the 2 h oil/water emulsion filtration, suggesting no oil fouling occurred on the membrane at all. At a higher initial permeate flux under a higher TMP, such as that of 0.12 MPa, the permeate flux was actually observed to decline with the filtration time. This was probably due to the higher feed pressure and hence higher convective flow that may have brought more oil to the membrane than it can reject and carry away by its cross-flow. In summary, the developed novel hollow fibre membrane may be able to completely avoid oil fouling under a properly selected operation condition (i.e. TMP).

In more details, it can be found that under the low TMPs, such as 0.04 MPa, there was almost no decay in the permeate flux at all. The permeate flux only declined slowly from 49.5 to 45.9 l m^−2^ h^−1^ at 0.06 MPa and from 58.4 to 54.3 l m^−2^ h^−1^ at 0.08 MPa, with RFD at about 7.2% and 7.0% of their initial fluxes, respectively. However, the permeate flux decayed from 68.9 to 59.0 l m^−2^ h^−1^ at 0.10 MPa and from 78.6 to 65.8 l m^−2^ h^−1^ at 0.12 MPa, with the RFD increased to 17.8% and 20.4%, respectively. Others also reported a faster flux decline at higher TMP than that at lower TMP [39]. In consideration of the performance results shown in figure 3*b*, the prepared hollow fibre membrane may be best operated at the TMP of 0.10 MPa or below.

The concentrate flow or cross-flow rate was also examined for its influence on the permeate flux. As shown in figure 3*c*, under the three different cross-flow rates investigated (30.3, 68.1 and 106.0 l h^−1^), the permeate fluxes all showed some decrease after 2 h filtration, but relatively insignificant with the RFDs at 20.4%, 17.8% and 14.5%, respectively, even though the test was conducted at a relatively higher TMP of 0.10 MPa. Although a higher cross-flow or concentrate flow appeared to reduce the oil fouling and hence the RFD, it may also mean more consumption of the process water and a lower water productivity. Since the novel membrane was oil-resistant and the increase of cross-flow rate did not seem to proportionally reduce the decline of the membrane flux, the cross-flow of 68.1 l h^−1^ has been selected as a choice for further experiments in the following sections discussed in this study.

### 3.3. Filtration of oil/water emulsion

**Figure 4.**
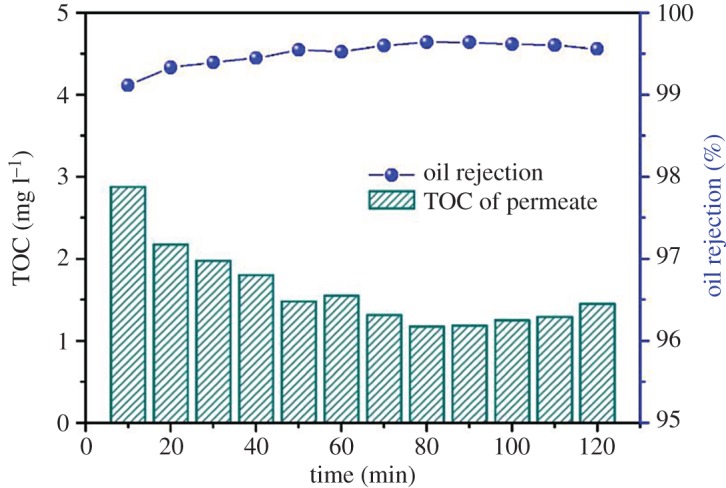
Variations of TOC in permeate and the TOC removal efficiency during the oil/water emulsion filtration.

As discussed in previous section, system operation conditions, i.e. at 25°C, TMP of 0.10 MPa, and cross-flow rate of 68.1 l h^−1^, and the oil/water emulsion at *c*_0_ = 400 mg l^−1^, were used to test the prepared hollow fibre membrane for oil separation efficiency and the performance in repeated usage of multiple cycles. Figure 4 shows the results of oil concentrations in permeate and oil rejection rates versus filtration time in terms of TOC values. It can be found that the prepared hollow fibre membrane showed very good oil/water separation performance. The TOC value of the permeate generally decreased along with the filtration time and, at all times, was maintained in the range between 1 and 3 mg l^−1^, with the oil rejection efficiency being always above 99% during the entire filtration process. This can be considered as an outstanding performance because many organic membranes are not able to be used directly and favourably for oil/water separation, especially at such a high feed oil concentration. Hence, the developed hollow fibre membrane has a great potential for engineering applications in oily wastewater treatment.

**Figure 5.**
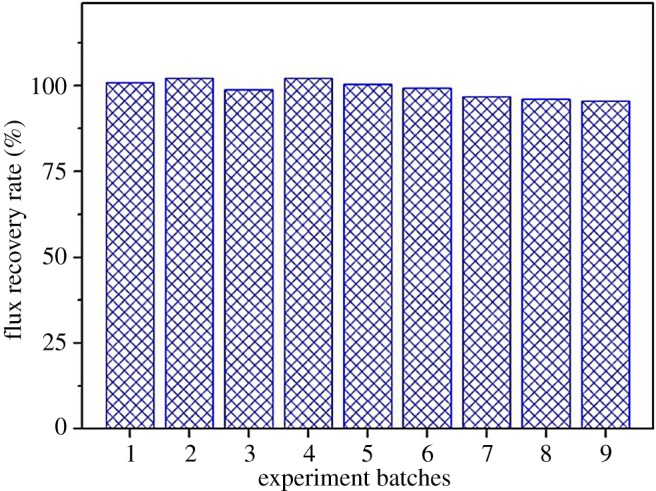
Permeate flux recovery of the prepared hollow fibre membrane during nine cycles of filtration tests.

It was also found that, under the above selected operation conditions, the hollow fibre membrane can achieve a very high flux recovery by simply using the DI water flushing, without any consumption of chemical agents. As shown in figure 5, the flux recovery rate was at a very high rate (greater than 95%) in the nine cycles of filtration experiments, each followed by a simple water flushing cleaning of the membrane. The consistent high flux recovery rates after repeated usages indicated that the prepared hollow fibre membrane indeed possessed an excellent ability in oil fouling resistance, which can be beneficially and effectively applied into practical applications in oil/water separation or oily wastewater treatment.

### 3.4. Treatment of real oily wastewater samples

In addition to the above studies on synthetic oil/water emulsion samples, the prepared hollow fibre membrane was also tested for some simulated or actual industrial oily wastewater, including those for oilfield effluent, palm oil plant effluent and mechanical cutting factory oily wastewater. All the wastewater samples were directly filtered by the prepared membrane module without any pretreatment. The concentration of oils in the feed and permeate was quantified by COD analysis.

**Figure 6.**
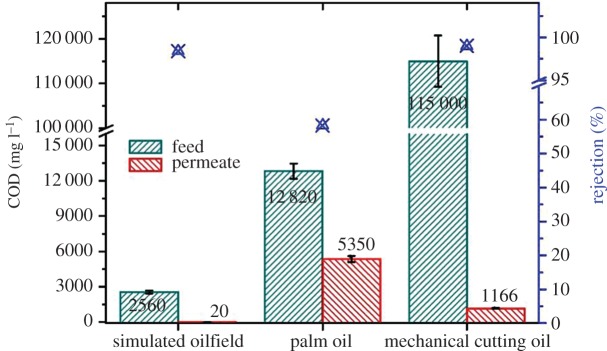
The COD removal and rejection rate of different oily wastewaters.

As shown in figure 6, for the simulated oilfield effluent sample (prepared from a mixture of crude oil, SDBS and inorganic salts), the feed had COD at 2560 mg l^−1^ and, after direct filtration, the COD of the permeate was reduced to 20 mg l^−1^, achieving a 98.4% rejection rate. For the real palm oil wastewater sample taken from a plant in Malaysia (figure 6, middle), it contained oil and other dissolved organic materials, with a COD up to 12 820 mg l^−1^. After the direct filtration with the prepared hollow fibre membrane module, approximately 58.3% of the COD was removed, together with a turbidity (in nephelometric turbidity units (NTU)) reduction of 97.2%. The oil emulsion effluent from a mechanical cutting plant in Jiangsu province of China had a much higher oil concentration (in COD) at as high as 115 000 mg l^−1^. From the direct filtration, an oil rejection rate up to 99.0% was obtained (figure 6, right).

**Figure 7.**
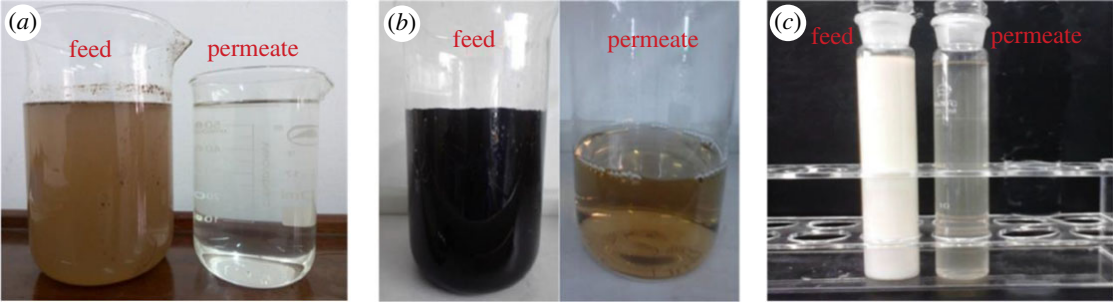
Visual comparison of the feed and permeate of different oily wastewater samples. (*a*) Simulated oilfield wastewater, (*b*) palm oil wastewater and (*c*) mechanical cutting oil wastewater.

A comparison in the visual observation of the feed and permeate samples from the membrane filtration system is shown in figure 7. Aesthetically, there are significant differences in the feed and the permeate samples. The results in this part of the study further support that the prepared hollow fibre membrane can have a wide adaptability to be used for a large range of feed oil concentrations, and for different types of oils and industries.

### 3.5. Continuous operation test

**Figure 8.**
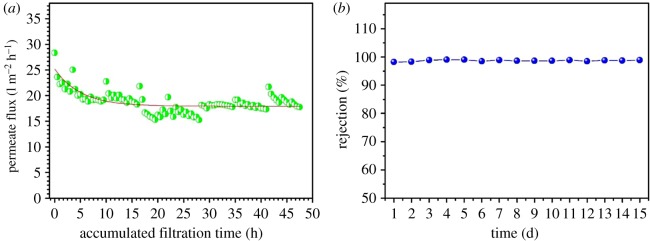
The permeate flux (*a*) and the oil rejection rate (*b*) of simulated palm oil wastewater under a long period of operation.

The prospect of the hollow fibre membrane for engineering application was also further investigated in a longer period of 15 days, with the prepared palm oil wastewater (TOC concentration at approx. 330 mg l^−1^, as shown in electronic supplementary material, table S9). Owing to the limitation of the test condition and set-up scale, the run time was made 4 h per day, and a simple water flushing (1 min) was provided after every 20 min filtration during the test. Figure 8*a* shows the permeate flux versus the accumulated filtration time. It can be found that the flux dropped from 28.3 to 19.1 l m^−2^ h^−1^ in the first 5 h or so, but thereafter the permeate flux remained almost constant at approximately 19.1 l m^−2^ h^−1^ throughout the rest of the test (TMP of 0.1 MPa). Within all the tests in the 15 days, the oil rejection rate was quite stable at approximately 99.0% (in terms of TOC), as shown in figure 8*b*. These results indicate that the prepared hollow fibre membrane can work well in prolonged operation, further supporting the potential of the prepared hollow fibre membrane for real filtration application to treat a large amount of oily wastewater.

## 4. Conclusion

The triblock copolymer additive AP can be successfully used with PVDF to prepare blend hollow fibre membranes. With the increase in the ratio of AP : PVDF from 0 : 10 to 2 : 8, the obtained membrane became more hydrophilic and oleophobic. The blend membrane with AP : PVDF at 1 : 9 was favourably selected in this study in considering its surface property, pore structure, water flux and strength. Various filtration tests were carried out with the prepared hollow fibre membrane module. It was found that the permeate flux of the membrane generally increased with the TMP, feed temperature and the cross-flow rate, with similar performance behaviours during water or oil/water emulsion filtration, largely attributed to its oil fouling resistant feature. When the membrane was operated at relatively low permeate flux, the flux can be maintained almost constant throughout the filtration process. Under an optimized operation condition, that is, at 25°C, TMP of 0.10 MPa and cross-flow flow of 68.1 l h^−1^, the hollow fibre membrane showed excellent oil/water separation performance. In terms of TOC, at feed oil concentration of 400 mg l^−1^, the oil/water emulsion, after the membrane direct filtration, had very low concentration in permeate, in the range of 1–3 mg l^−1^, and achieved greater than 99% oil rejection. The flux recovery rate, by simple water flushing, was maintained at a very high rate (greater than 95%) after nine filtration cycles tested. More importantly, the prepared membrane demonstrated a wide adaptability and high efficiency when directly applied into the treatment of various simulated or real industrial oily wastewater that had very high oil concentration, or different types of oil or from different industries. In addition, a prolonged period of filtration tests within 15 days showed consistent permeate flux of approximately 19.1 l m^−2^ h^−1^, and 99% of oil rejection performance. The study provides a valuable reference to the practical application of the developed hollow fibre membrane that has both hydrophilic and oleophobic surface properties.

## Supplementary Material

Supplementary material

## Data accessibility

The datasets supporting this article have been uploaded as part of the electronic supplementary material.
