# Supplementary material for: Application of a triblock copolymer additive modified polyvinylidene fluoride membrane for effective oil/water separation
Source: R Soc Open Sci. 2018 May 9;5(5):171979. doi: 10.1098/rsos.171979 (PMC5990766; doi:10.1098/rsos.171979)
Supplement: Supplementary material [file rsos171979supp1.doc]

**Supplementary material**

**Application of a triblock copolymer additive modified polyvinylidene
ﬂuoride membrane for effective oil/water separation**

S. S. Shen*^a,b,c^*, K.P. Liu*^a^*, J. J. Yang*^a,b,c^*, Y. Li*^a^*, R. B. Bai*^a,b,c^* *, X. J. Zhou^a,b,c^

^a^ Center for Separation and Purification Materials & Technologies,

^b^ Suzhou Key Laboratory of Separation and Purification Materials & Technologies,

^c^ Jiangsu Collaborative Innovation Center for Technology and Material of Water Treatment,

Suzhou University of Science and Technology, 1 Kerui Road, Suzhou 215009, China

** Author for all correspondence: brb998@outlook.com (R.B. Bai)*

**Fig.S1.** The size distributions of oil droplets in the sample of concentration of 400 mg·L^-1^.

Experimental data summary for each figures in the article are summarized as follows**：**

**1. Figure 1a-d**

| **Table S1** The composition and properties of the hollow fiber membranes | | | |
| --- | --- | --- | --- |
| membranes | **M0** | **M1** | **M2** |
| ratio of PVDF/AP | **10:0** | **9:1** | **8:2** |
| water contact angle (°) | 62.58±4.02 | 44.63±1.29 | 35.94±1.60 |
| oil contact angle (°) | 11.91±2.38 | 59.06±3.10 | 72.06±3.89 |
| maximum pore size (μm) | 0.717±0.05 | 1.205±0.05 | 1.981±0.11 |
| permeation flux (L·m^-2^·h^-1^) | 266.6±8.9 | 342.6±15.1 | 497.6±23.2 |
| tensile strain (%) | 92.36±3.23 | 80.45±4.10 | 65.94±1.39 |
| tensile stress (MPa) | 1.33±0.03 | 1.07±0.05 | 0.75±0.02 |

2. Figure 3a

| **Table S2** The permeate flux of prepared membrane under different feed temperature. | | | | | | |
| --- | --- | --- | --- | --- | --- | --- |
|  | DI water flux ((L·m^-2^·h^-1^) | | | Oil/water emulsion flux ((L·m^-2^·h^-1^) | | |
|  | 0.06 MPa | 0.08 MPa | 0.10 MPa | 0.06 MPa | 0.08 MPa | 0.10 MPa |
| 20 ^o^C | 32.71 | 43.13 | 52.10 | 31.82 | 43.34 | 52.74 |
| 25 ^o^C | 36.72 | 47.50 | 59.11 | 36.97 | 47.9 | 61.94 |
| 30 ^o^C | 41.15 | 55.06 | 65.15 | 40.74 | 52.52 | 66.90 |
| 35 ^o^C | 48.07 | 61.51 | 72.57 | 48.28 | 57.33 | 71.23 |
| 40 ^o^C | 54.48 | 67.03 | 80.18 | 51.91 | 62.35 | 76.18 |

**3. Figure 3b**

| **Table S3** The permeate flux versus running time of prepared membrane for oil/water emulsion. | | | | | |
| --- | --- | --- | --- | --- | --- |
| time | Permeate flux (L·m^-2^·h^-1^) | | | | |
|  | 0.04 MPa | 0.06 MPa | 0.08 MPa | 0.10 MPa | 0.12 MPa |
| 5 min | 34.19 | 49.5 | 58.42 | 68.87 | 78.64 |
| 10 min | 34.19 | 48.68 | 58.1 | 67.97 | 76.87 |
| 15 min | 34.35 | 48.15 | 57.78 | 66.75 | 76.22 |
| 20 min | 34.32 | 47.92 | 57.56 | 66.55 | 75.45 |
| 25 min | 34.26 | 47.71 | 57.48 | 66.25 | 74.82 |
| 30 min | 34.22 | 47.12 | 57.02 | 66.22 | 73.35 |
| 40 min | 34.27 | 46.86 | 56.49 | 66.18 | 72.86 |
| 50 min | 34.04 | 47.01 | 56.12 | 65.35 | 71.2 |
| 60 min | 34.08 | 46.86 | 56.55 | 63.54 | 70.69 |
| 70 min | 33.97 | 46.68 | 56.29 | 62.47 | 70.68 |
| 80 min | 34.06 | 46.24 | 56.69 | 63.05 | 69.46 |
| 90 min | 34.06 | 45.81 | 56.01 | 62.45 | 67.99 |
| 100 min | 33.94 | 46.33 | 56.2 | 62.39 | 66.83 |
| 110 min | 33.98 | 44.87 | 55.9 | 62.23 | 65.54 |
| 120 min | 34 | 45.92 | 54.32 | 59.03 | 65.75 |

4. Figure 3c

| **Table S4** The permeate fluxes of oil/water emulsion under different concentrate flows. | | | |
| --- | --- | --- | --- |
| time | Permeate flux (L·m^-2^·h^-1^) | | |
|  | 30.28 L·h^-1^ | 68.13 L·h^-1^ | 105.98 L·h^-1^ |
| **0 min** | **62.61** | **71.85** | **78.62** |
| 5 min | 60.64 | 68.87 | 78.3 |
| 10 min | 58.97 | 67.97 | 77.85 |
| 15 min | 55.07 | 66.75 | 76.98 |
| 20 min | 54.72 | 66.55 | 76.24 |
| 25 min | 53.88 | 66.25 | 75.56 |
| 30 min | 52.13 | 66.22 | 74.71 |
| 40 min | 52.53 | 66.18 | 73.19 |
| 50 min | 51.94 | 65.35 | 71.83 |
| 60 min | 51.25 | 63.54 | 71.75 |
| 70 min | 50.47 | 62.47 | 69.68 |
| 80 min | 50.58 | 63.05 | 69.22 |
| 90 min | 49.82 | 62.45 | 68.71 |
| 100 min | 50.43 | 62.39 | 68.89 |
| 110 min | 49.51 | 62.23 | 67.58 |
| **120 min** | **49.86** | **59.03** | **67.19** |

**5. Figure 4**

| **Table S5** The TOC concentration and oil rejection of permeate *versus* running time. | | |
| --- | --- | --- |
| time (min) | TOC concentration (mg/L) | Oil rejection rate (%) |
| 10 | 2.874 | 99.12 |
| 20 | 2.175 | 99.33 |
| 30 | 1.975 | 99.39 |
| 40 | 1.801 | 99.45 |
| 50 | 1.477 | 99.55 |
| 60 | 1.548 | 99.52 |
| 70 | 1.314 | 99.60 |
| 80 | 1.172 | 99.64 |
| 90 | 1.186 | 99.64 |
| 100 | 1.25 | 99.62 |
| 110 | 1.29 | 99.60 |
| 120 | 1.45 | 99.55 |

6. Figure 5

| **Table S6** The permeate flux recovery of the prepared hollow fiber membrane. | | | | | | | | | |
| --- | --- | --- | --- | --- | --- | --- | --- | --- | --- |
| Experiment batches | 1 | 2 | 3 | 4 | 5 | 6 | 7 | 8 | 9 |
| Flux recovery rate (%) | 100.7 | 102.1 | 98.9 | 102.2 | 100.4 | 99.3 | 96.8 | 96.0 | 95.4 |

7. Figure 6

| **Table S7** The rejection rate of different oily wastewater. | | | |
| --- | --- | --- | --- |
| Oily wastewater | COD (mg/L) | | Rejection rate (%) |
|  | feed | permeate |  |
| *simulated oilfield* | 2,560 | 20 | 98.4 |
| *palm oil* | 12,820 | 5,350 | 58.3 |
| *mechanical cutting oil* | 115,000 | 1,166 | 99.0 |

**8. Figure 8a**

| **Table S8** The permeate flux of mimic palm oil wastewater under continuous operation. | | | |
| --- | --- | --- | --- |
| Operation time /h | Water flux /L·m^-2^·h^-1^ | Operation time /h | Water flux/L·m^-2^·h^-1^ |
| 0 | 28.29787234 | 11.5 | 20.12553191 |
| 0.5 | 23.5893617 | 12 | 19.4106383 |
| 1 | 22.24042553 | 12.5 | 20.10851064 |
| 1.5 | 22.52765957 | 13 | 19.24042553 |
| 2 | 21.24042553 | 13.5 | 19.19148936 |
| 2.5 | 22.2787234 | 14 | 18.64042553 |
| 3 | 20.80851064 | 14.5 | 19.4212766 |
| 3.5 | 25.03191489 | 15 | 18.84255319 |
| 4 | 21.22340426 | 15.5 | 18.66808511 |
| 4.5 | 19.99361702 | 16 | 18.25106383 |
| 5 | 20.27446809 | 16.5 | 21.8212766 |
| 5.5 | 19.24042553 | 17 | 19.22553191 |
| 6 | 19.31702128 | 17.5 | 16.66170213 |
| 6.5 | 18.85744681 | 18 | 16.34255319 |
| 7 | 19.73617021 | 18.5 | 15.87446809 |
| 7.5 | 19.1212766 | 19 | 15.70212766 |
| 8 | 19.19148936 | 19.5 | 15.3106383 |
| 8.5 | 19.07234043 | 20 | 16.26170213 |
| 9 | 18.82978723 | 20.5 | 15.84255319 |
| 9.5 | 19.12978723 | 21 | 17.26382979 |
| 10 | 22.75531915 | 21.5 | 16.41489362 |
| 10.5 | 20.37659574 | 22 | 19.6893617 |
| 11 | 19.5 | 22.5 | 16.98297872 |
|  |  |  |  |
| Operation time /h | Water flux /L·m^-2^·h^-1^ | Operation time /h | Water flux /L·m^-2^·h^-1^ |
| 23.5 | 17.7212766 | 35.5 | 19.14893617 |
| 24 | 16.81914894 | 36 | 18.33404255 |
| 24.5 | 17.26382979 | 36.5 | 18.56808511 |
| 25 | 16.27234043 | 37 | 17.99787234 |
| 25.5 | 16.82340426 | 37.5 | 18.26382979 |
| 26 | 16.05531915 | 38 | 17.75957447 |
| 26.5 | 16.44042553 | 38.5 | 18.12553191 |
| 27 | 15.79361702 | 39 | 17.60851064 |
| 27.5 | 15.83191489 | 39.5 | 18.0106383 |
| 28 | 15.26170213 | 40 | 17.49574468 |
| 28.5 | 18.11702128 | 40.5 | 17.48297872 |
| 29 | 17.90425532 | 41 | 17.34468085 |
| 29.5 | 17.50851064 | 41.5 | 21.67446809 |
| 30 | 18.26382979 | 42 | 20.32765957 |
| 30.5 | 18.09148936 | 42.5 | 19.5893617 |
| 31 | 18.08723404 | 43 | 19.13404255 |
| 31.5 | 18.29361702 | 43.5 | 18.61276596 |
| 32 | 18.25106383 | 44 | 19.64042553 |
| 32.5 | 18.22340426 | 44.5 | 19.16382979 |
| 33 | 18.11702128 | 45 | 18.72765957 |
| 33.5 | 18.06170213 | 45.5 | 18.22340426 |
| 34 | 17.91702128 | 46 | 18.79574468 |
| 34.5 | 17.8 | 46.5 | 18.29361702 |
| 35 | 19.14893617 | 47 | 17.85106383 |

**9. Figure 8b**

**Table S9** The oil rejection rate of mimic palm oil wastewater under continuous filtration (15 days) *^a^*.

| filtration day | Feed concentration（mg/L） | Permeate concentration（mg/L） | Rejection rate（%） |
| --- | --- | --- | --- |
| 1 | 308 | 5.45 | 98.2 |
| 2 | 324 | 5.6 | 98.3 |
| 3 | 315 | 3.38 | 98.9 |
| 4 | 330 | 3.32 | 99.0 |
| 5 | 319 | 3.15 | 99.0 |
| 6 | 329 | 5.01 | 98.5 |
| 7 | 316 | 3.61 | 98.9 |
| 8 | 330 | 4.53 | 98.6 |
| 9 | 319 | 4.42 | 98.6 |
| 10 | 326 | 4.54 | 98.6 |
| 11 | 325 | 3.59 | 98.9 |
| 12 | 317 | 4.78 | 98.5 |
| 13 | 335 | 3.87 | 98.8 |
| 14 | 347 | 4.39 | 98.7 |
| 15 | 336 | 3.58 | 98.9 |
| *^a^*The feed and permeate concentration were recorded as the average TOC value of permeate on each day. | | | |
